# Causal association between metabolic syndrome and cholelithiasis: a Mendelian randomization study

**DOI:** 10.3389/fendo.2023.1180903

**Published:** 2023-06-09

**Authors:** Qi Zhu, Yawei Xing, Yunfeng Fu, Xiaqin Chen, Langyi Guan, Foqiang Liao, Xiaodong Zhou

**Affiliations:** Department of Gastroenterology, Digestive Disease Hospital, The First Affiliated Hospital of Nanchang University, Nanchang, China

**Keywords:** cholelithiasis, obesity, metabolic syndrome, abdominal obesity, Mendelian randomization

## Abstract

**Background:**

Metabolic syndrome (MetS) has been associated with digestive system diseases, and recent observational studies have suggested an association between MetS and cholelithiasis. However, the causal relationship between them remains unclear. This study aimed to assess the causal effect of MetS on cholelithiasis using Mendelian randomization (MR) analysis.

**Methods:**

Single nucleotide polymorphisms (SNPs) of MetS and its components were extracted from the public genetic variation summary database. The inverse variance weighting method (IVW), weighted median method, and MR-Egger regression were used to evaluate the causal relationship. A sensitivity analysis was performed to ensure the stability of the results.

**Results:**

IVW showed that MetS increased the risk of cholelithiasis (OR = 1.28, 95% CI = 1.13–1.46, P = 9.70E−05), and the weighted median method had the same result (OR = 1.49, 95% CI = 1.22–1.83, P = 5.68E−05). In exploring the causal relationship between MetS components and cholelithiasis, waist circumference (WC) was significantly associated with cholelithiasis. IVW analysis (OR = 1.48, 95% CI = 1.34–1.65, P = 1.15E−13), MR-Egger regression (OR = 1.62, 95% CI = 1.15–2.28, P = 0.007), and weighted median (OR = 1.73, 95% CI = 1.47–2.04, P = 1.62E−11) all found the same results.

**Conclusion:**

Our study indicated that MetS increases the incidence of cholelithiasis, especially in MetS patients with abdominal obesity. Control and treatment of MetS can effectively reduce the risk of gallstone formation.

## Introduction

Metabolic syndrome (MetS) is a common metabolic disorder that is usually composed of a group of abnormal conditions, including abdominal obesity, hypertension, hyperglycemia, an increased triglyceride (TG) level, and a decreased high-density lipoprotein cholesterol (HDL-C) level. The incidence of MetS has been increasing, and the affected population gradually tends to be younger. Studies (1, 2) have shown that the global incidence of MetS is 20% to 50% and reaches 50% in severely obese adolescents. MetS increases the risk of cardiovascular disease, fatty liver disease, and type 2 diabetes and is closely related to numerous digestive system diseases (3, 4).

Cholelithiasis is an increasingly common digestive system disease. Approximately 10%–20% of adults have cholelithiasis, and 20% of patients will develop complications (5–7), such as cholecystitis, cholangitis, pancreatitis, gallbladder perforation, and sepsis. Cholelithiasis and its complications increase the cost of health care, the economic burden on society, and even threaten people’s lives. Because both MetS and cholelithiasis are closely associated with type 2 diabetes (8, 9), their relationship has received widespread attention in recent years. Previous studies (10–12) have shown a significant association between MetS and cholelithiasis, and some studies (11, 12) have even hypothesized that cholelithiasis should be a component of MetS. However, these studies are retrospective, and there is an inevitable bias. The association between MetS and cholelithiasis in retrospective studies is susceptible to confounding factors such as small sample size, limited follow-up time, and reverse causality, which may lead to biased conclusions (13).

Mendelian randomization is a novel statistical method that minimizes confounding effects and backward effects, providing reliable evidence for a causal relationship between the metabolic syndrome and cholelithiasis (14, 15). If exposure variables (MetS) affect outcome variables (cholelithiasis), then genetic variation in MetS traits should affect the occurrence of cholelithiasis to some extent when pleiotropy is excluded. Since the causal relationship between MetS and cholelithiasis has not been fully elucidated, in this study we applied MR analysis to assess the causal impact of MetS on cholelithiasis and to provide constructive suggestions for the prevention of cholelithiasis.

## Materials and methods

### Overview of Mendelian randomization analysis

**Figure 1:**
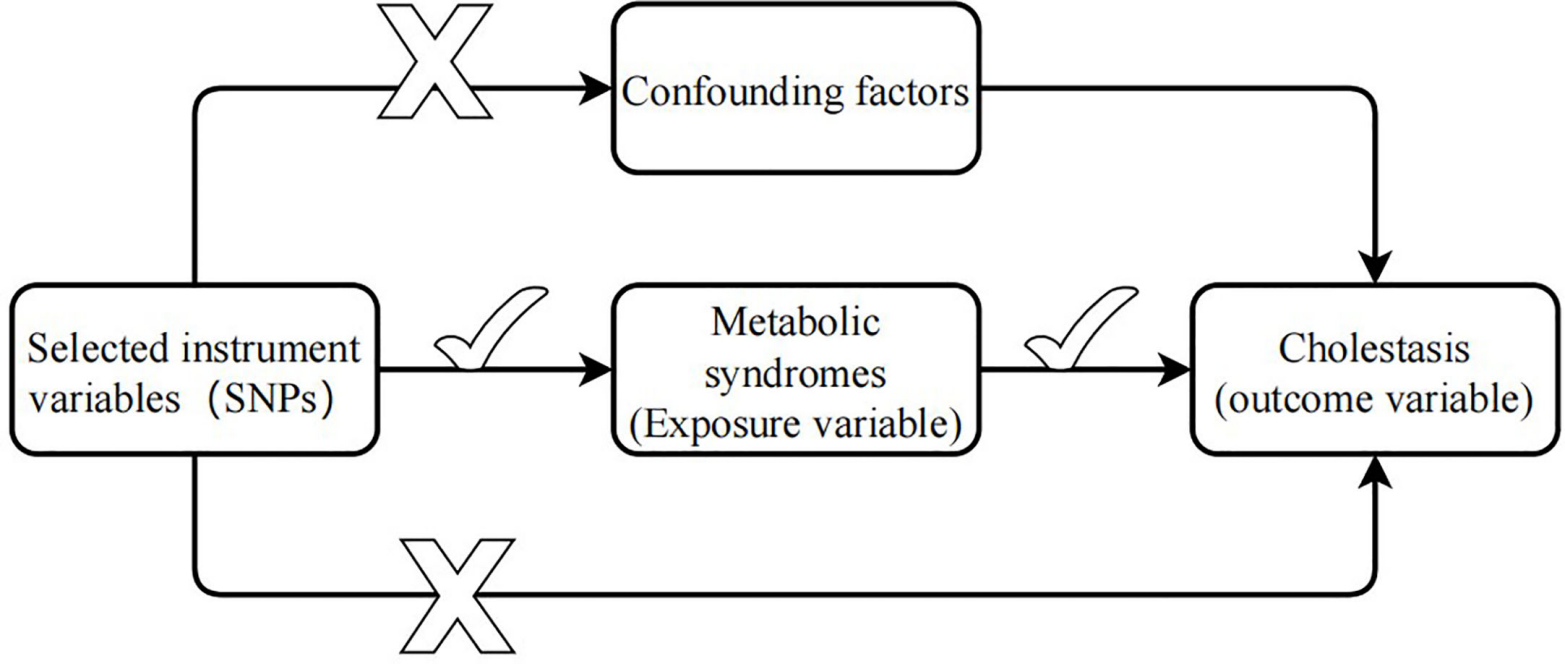
Three hypotheses of Mendelian randomization.

Mendelian randomization (MR) analysis, which uses single nucleotide polymorphisms (SNPs) as instrumental variables (IVs) to control for the effects of confounding factors, is increasingly used to infer causal relationships between risk factors and diseases (16). MR analysis uses summary statistics from a genome-wide association study (GWAS) with a large number of genetic variants to extract SNPs associated with exposure (e.g., MetS) and outcome variables (e.g., cholelithiasis). Because genotypes are determined before birth and follow random assortment during meiosis (17), MR analysis can effectively overcome confounding factors to determine the causal relationship between exposure and outcome. IVs in MR analysis need to satisfy three core assumptions (18) (1): The selected genetic instrument needs to be strongly correlated with the exposure variable (2); there is no association between the genetic instrument and any confounding factors; and (3) the genetic instrument and the outcome have no common cause and affect the outcome only through the exposure variable (**Figure 1**).

### Instrumental variables associated with MetS and components

We extracted IVs from the latest GWAS summary-level data and performed a two-sample MR analysis to explore the causal effect of MetS on cholelithiasis. For metabolic syndrome, genetic instruments are based on the latest data from the Complex Trait Genetics Lab (CTG), which contains 461,920 valid subjects of European ancestry and identifies genetic variation in metabolic syndrome through the structural equation modeling method (19). IVs are determined by the following criteria (1): SNPs with genome-wide significance (*P <*5 × 10^−8^) (2); we use the PLINK algorithm to remove linkage disequilibrium (r^2^ threshold = 0.001 and clumping distance = 10,000 kb) (3); removal of SNPs associated with confounding factors (4); harmonizing processes were conducted to exclude ambiguous and palindromic SNPs (5); removal of SNPs containing pleiotropy. Therefore, a total of 155 SNPs were included in the Mendelian randomization analysis in our study. **Table 1** shows more details of the instrumental variables, and an F-statistic greater than 10 indicates that the instrumental variables have a strong potential to predict cholelithiasis (20).

**Table 1:** Overview of data sources and strength analysis.

Exposure	Ancestry	Sample size (case/control)	Data sources	R2 (%)	F-statistic	Web source
Metabolic syndrome	European	461,902	Jorim J. Tielbeek et al.	0.91	38.27	https://ctg.cncr.nl/software/summary_statistics
FBG	European	58,074	Alisa K Manning et al.	2.67	41.50	https://gwas.mrcieu.ac.uk/datasets/ebi-a-GCST005186/
WC	European	462,166	MRC-IEU	0.95	41.73	https://gwas.mrcieu.ac.uk/datasets/ukb-b-9405/
Hypertension	European	119,731/343,202	MRC-IEU	1.00	46.41	https://gwas.mrcieu.ac.uk/
HDL-C	Mixed (96% European)	187,167	GLGC	1.70	51.52	https://gwas.mrcieu.ac.uk/datasets/ieu-a-299/
Triglycerides	Mixed (96% European)	177,861	GLGC	1.68	49.73	https://gwas.mrcieu.ac.uk/datasets/ieu-a-302/
Cholelithiasis	European	19,023/195,144	FinnGen Consortium	/	/	https://www.finngen.fi/fi

MetS, metabolic syndrome; FBG, fasting blood glucose; WC, waist circumference; TG, triglycerides; HDL-C, high-density lipoprotein cholesterol.

Genetic variation data for metabolic syndrome components were extracted from the comprehensive GWAS and were all screened according to previous criteria, and the F-statistic of all genetic instruments is greater than 10 (**Table 1**). We extracted genetic instruments for fasting blood glucose (FBG) from the database, which contains 58,074 participants of European ancestry, and the data were adjusted for BMI (21). After screening, 18 SNPs were strongly associated with fasting glucose and included in the analysis.

We extracted genetic instruments for waist circumference (WC) from the Medical Research Council Integrative Epidemiology Unit (MRC-IEU) UK Biobank GWAS Pipeline, which included 462,166 subjects with European ancestry, and 304 SNPs were included in the analysis after screening.

For hypertension, also extracted from the MRC-IEU UK Biobank pipeline, the data sample consisted of 119,731 cases and 343,202 controls; all participants were of European ancestry; and 83 SNPs were included in the analysis after screening.

For HDL-C and TG, summary statistics were obtained from the Global Lipids Genetics Consortium (GLGC), which consists of multiracial participants, but 96% of them are of European ancestry (22). After a series of screenings, 63 SNPs and 40 SNPs, respectively, were finally included in the analysis.

### GWAS summary-level data for cholelithiasis

We extracted genetic variation data for cholelithiasis from the FinnGen Consortium. The summary-level statistics are from a large study involving 19,023 cases and 195,144 controls, all of European ancestry. In this large study, cases of cholelithiasis were diagnosed based on the International Classification of Diseases, 10th Revision (ICD-10) and code K80 definitions (**Table 1**). Adjustments were also made for sex age and genotyping batches using strict SNP inclusion criteria (MAF >1%).

### MR analysis method

In our study, we used three methods, including inverse variance weighting (IVW), MR-Egger regression, and weighted median, respectively, applied to the two-sample MR analysis to estimate the causal relationship between MetS and cholelithiasis. Our primary analysis was estimated with the use of the IVW method, which is a method of combining the Wald ratio of each SNP for the outcome to obtain a pooled causal estimate (23). The MR-Egger regression allows testing for unbalanced pleiotropy and considerable heterogeneity (24). The weighted median method provides consistent effect estimates when at least half of the weighted variance provided by horizontal pleiotropy is valid (25). MR-Egger and weighted median methods were used to supplement IVW estimation because these methods can provide more robust estimates in a wider range of scenarios but are less effective. In addition, sensitivity tests were performed to ensure the stability of the analysis results. Sensitivity analysis methods included Cochran’s Q test, funnel pot, leave-one-out (LOO) analyses, and MR-Egger intercept tests. Specifically, heterogeneity was observed with a P-value of less than 0.05 by Cochran’s Q test. The intercept derived from the MR-Egger regression assessed horizontal pleiotropy. LOO tests are used to determine whether a single SNP determines the inference of causal association by repeating the IVW analysis by dropping each SNP associated with exposure in turn. MR-PRESSO was used to detect outliers and to assess and correct for horizontal pleiotropy (26).

### Statistical analysis

Bonferroni-corrected *P*-values of 0.008 (0.05/6 = 0.008) were used to determine statistical significance in the MR analysis; *P <*0.008 was considered statistically significant, and all *P*-values are two-tailed. MR estimates were presented as odds ratios (OR) and corresponding 95% confidence intervals (CI). R^2^ was calculated to represent the proportion of the variance of exposure factors explained by instrumental variables. F-statistics were calculated to represent the strength of the association between instrumental variables and exposure factors (**Table 1**). All analyses were performed by the packages TwoSampleMR (version 0.5.6) in R software (version 4.2.2).

## Results

### Causal effects of metabolic syndrome and cholelithiasis

**Figure 2:**
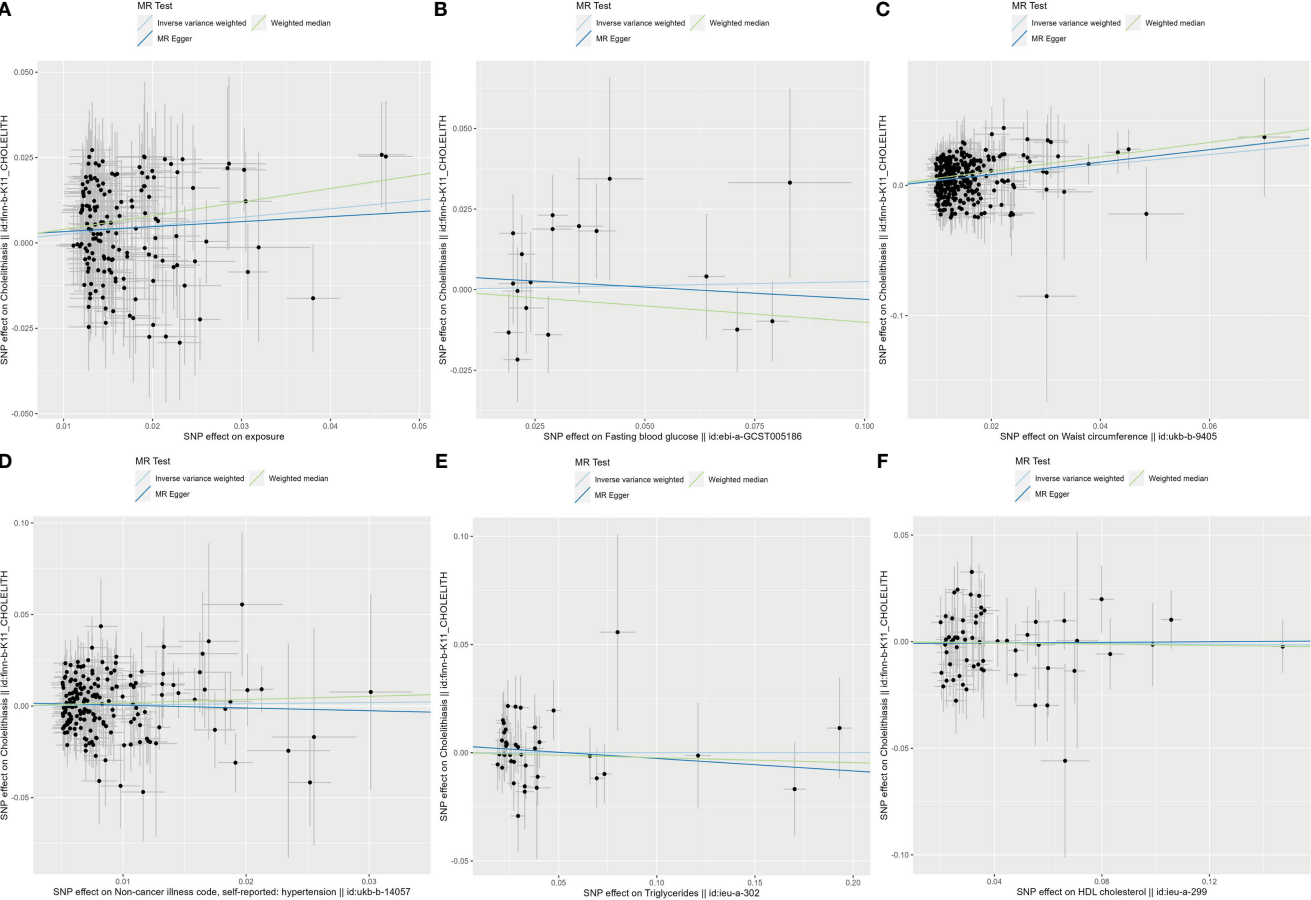
The scatter plots for MetS and its components. **(A)** Scatter plot of MetS and cholelithiasis; **(B)** Scatter plot of FBG and cholelithiasis; **(C)** Scatter plot of WC and cholelithiasis; **(D)** Scatter plot of hypertension and cholelithiasis; **(E)** Scatter plot of TG and cholelithiasis; **(F)** Scatter plot of HDL-C and cholelithiasis. MetS, metabolic syndrome; FBG, fasting blood glucose; WC, waist circumference; TG, triglycerides; HDL-C, high-density lipoprotein cholesterol.

After the SNP clumping step using the PLINK algorithm, we removed SNPs associated with the outcome and SNPs with palindromic structure, and finally, 155 SNPs closely related to MetS were included in the final MR analysis. The findings of MR analysis and sensitivity analysis are presented in **Table 2**, and scatter and forest plots are presented in **Figures 1**, **2**. They both indicate a positive causal relationship between MetS and gallstone disease. The results of IVW analysis (OR = 1.28, 95% CI = 1.13–1.46, *P* = 9.70E−05) and weighted median analysis (OR = 1.49, 95% CI = 1.22–1.83, *P* = 5.68E−05) showed that MetS increased the risk of cholelithiasis. Although not statistically significant, the MR-Egger results (OR = 1.16, 95% CI = 0.77–1.75, *P* = 0.486) still suggest that MetS raises the risk of cholelithiasis (**Table 2**). The MR-Egger (*P* = 0.529) and IVW (*P* = 0.0.546) *P*-values of the Cochran Q-test indicated no heterogeneity in our results, and no significant MR-Egger intercept values (intercept = 0.00183; *P* = 0.606) were observed. Furthermore, the MR-PRESSO (global test P-value = 0.545) results also showed no directional pleiotropy in our results. The funnel plots were symmetrical (**Supplementary Figure S1**), and in the leave-one-out sensitivity analysis, no single SNP was observed to significantly affect the overall results (**Supplementary Figure S2**), all of which support the stability of the results of our MR analysis.

**Table 2:** Association of MetS and its components with cholelithiasis in MR analysis.

Exposure	Method	OR (95%CI)	*P*-value	Cochran Q test *P*-value	Egger_intercept	*P*-Egger_intercept	MR-PRESSO *P*-value
Metabolic syndrome							0.545
	MR Egger	1.16 (0.77–1.75)	0.486	0.529	0.00183	0.606	
	Weighted median	1.49 (1.22–1.83)	5.68E−05				
	Inverse variance weighted	1.28 (1.13–1.46)	9.70E−05	0.546			
FBG							0.306
	MR Egger	0.93 (0.65–1.33)	0.688	0.270	0.00458	0.536	
	Weighted median	0.90 (0.71–1.15)	0.420				
	Inverse variance weighted	1.03 (0.86–1.23)	0.787	0.303			
WC							0.942
	MR Egger	1.62 (1.15–2.28)	0.007	0.935	−0.00130	0.614	
	Weighted median	1.73 (1.47–2.04)	1.62E−11				
	Inverse variance weighted	1.48 (1.34–1.65)	1.15E−13	0.939			
Hypertension							0.798
	MR Egger	0.86 (0.45–1.67)	0.659	0.786	0.00194	0.497	
	Weighted median	1.20 (0.80–1.78)	0.377				
	Inverse variance weighted	1.07 (0.84–1.36)	0.604	0.794			
TG							0.928
	MR Egger	0.94 (0.80–1.11)	0.502	0.928	0.00311	0.401	
	Weighted median	0.98 (0.85–1.13)	0.756				
	Inverse variance weighted	1.00 (0.91–1.10	0.996	0.930			
HDL							0.616
	MR Egger	1.01 (0.87–1.17)	0.924	0.582	−0.0009	0.788	
	Weighted median	0.99 (0.87–1.12)	0.819				
	Inverse variance weighted	0.99 (0.92–1.07)	0.803	0.615			

MetS, metabolic syndrome; FBG, fasting blood glucose; WC, waist circumference; TG, triglycerides; HDL-C high-density lipoprotein cholesterol.

### Causal relationship between components of metabolic syndrome and cholelithiasis

**Figure 3:**
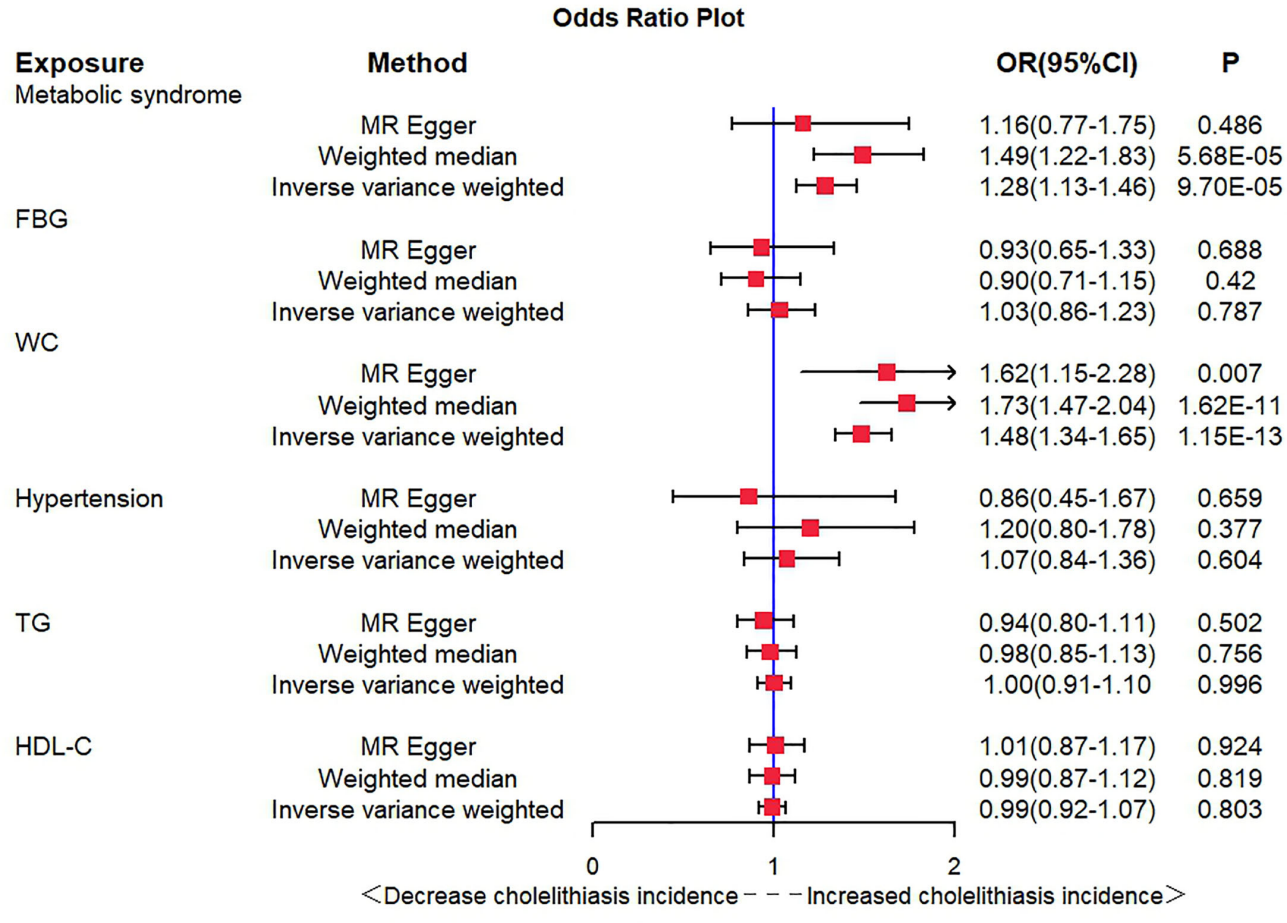
Odds ratio plot for MetS and its components.

In exploring the causal relationship between components of the metabolic syndrome and gallstone disease, we used 18 FBG variants, 304 WC variants, 183 hypertension variants, 63 HDL-C variants, and 40 TG variants as genetic instruments for exposure factors, respectively, after excluding unavailable SNPs and palindromic SNPs from the summary-level dataset. The results of the MR analysis showed a causal relationship between WC and cholelithiasis. The IVW method (OR = 1.48, 95% CI = 1.34–1.65, *P* = 1.15E−13), the MR-Egger method (OR = 1.62, 95% CI = 1.15–2.28, *P* = 0.007), and the weighted median method (OR = 1.73, 95% CI = 1.47–2.04, *P* = 1.62E−11) all suggest statistically significant results and in the same direction, suggesting that an increase in waist circumference raises the risk of cholelithiasis (**Table 2**). Similarly, the results were found in scatter plots (**Figure 1**) and forest plots (**Figure 2**). In addition, the Cochran Q test (IVW, *P* = 0.939; MR-Egger, *P* = 0.935) did not suggest significant heterogeneity, and no pleiotropy was observed with the MR-Egger intercept test (intercept = −0.00130; *P* = 0.614) or the MR-PRSSON test (Global Test *P-*value = 0.942). The funnel plots (**Supplementary Figure S1**) and leave-one-out method (**Supplementary Figure S2**) also reveal the reliability of the results. The remaining four components were not statistically significantly associated with cholelithiasis (**Table 2**). Although the P-values were not statistically significant, FBG, hypertension, and TG still tended to increase the risk of cholelithiasis, while HDL-C tended to decrease the risk (**Figure 3**).

## Discussion

To the best of our knowledge, this is the first study to assess the causality between MetS and its components and cholelithiasis on a large scale using MR analysis. The results showed that MetS and waist circumference were significantly associated with cholelithiasis. Sensitivity and pleiotropy analyses indicated that our results were stable.

A new study (27) found that MetS showed a strong association with the incidence of cholelithiasis. A cross-sectional study (12) has shown that the presence of MetS was associated with an increased risk of cholelithiasis, and WC, as a component of the metabolic syndrome, was the most important factor associated with the risk of cholelithiasis, which is consistent with our findings. Furthermore, in another study (10), a positive correlation was found between the number of metabolic syndrome components and the prevalence of cholelithiasis in men, while no such trend was observed in women with MetS. In contrast, the latest study (28) showed that the greater the number of metabolic components present, the higher the risk of cholelithiasis in both men and women. This difference may be attributed to bias due to confounding factors in observational studies. Notably, since MR was analyzed at the genetic level, our study minimized the effects of confounding factors such as the external environment, lifestyle, and dietary habits.

WC has been shown to be the best anthropometric measure of visceral adipose tissue mass with an approximate linear correlation and is a reliable indicator of abdominal obesity (29). Obesity is a cornerstone of MetS and an important risk factor for cholelithiasis. Several studies (30–32) have revealed a significant correlation between obesity and gallstone disease, and obesity is associated with the development of gallstone disease in patients with type 2 diabetes (31). This increased risk may be due to the presence of cholesterol, and obesity increases hepatic cholesterol secretion (30), leading to bile supersaturation and ultimately gallstone formation. In addition, Mendelian studies (33) have shown a causal relationship between elevated BMI and an increased risk of symptomatic cholelithiasis.

In our study, we screened 155 SNPs that were strongly associated with MetS, and our results showed a positive causal association between MetS and cholelithiasis in both IVW and weighted median analyses. In addition, in its component analysis, we also found that abdominal circumference significantly increased the occurrence of cholelithiasis. In contrast, FBG, hypertension, TG, and HDL-C were not causally associated with cholelithiasis, although some observational studies (34–38) suggest an epidemiological link between them. These results all imply that the causal association between MetS and cholelithiasis may be mediated by obesity.

Most previous studies have been retrospective, which inevitably produces confounding factors, and randomized controlled trials can be time- and resource-intensive and add to the research burden. Our study has several strengths: First, our use of an MR study design effectively avoids confounding factors and reverses causality measurement errors. Second, we used comprehensive GWAS summary-level data on the MetS and its components and therefore had high investigative power. Finally, we applied a series of sensitivity analyses to increase the credibility of our results.

But there are some limitations worth noting. Firstly, Europeans form a major part of our study, making it difficult to generalize the results to other ethnic groups with different lifestyles. Secondly, the dataset we used did not allow for stratified analysis, and future subgroup analyses should be conducted to further explore the causal relationship between MetS and different types of cholelithiasis.

## Conclusion

In conclusion, our study strengthens the evidence for a causal relationship between MetS and cholelithiasis. In clinical practice, the occurrence and development of cholelithiasis in patients with MetS should be considered. It is necessary to strengthen the management of MetS and timely screen for cholelithiasis, especially in obese patients with a large waist circumference.

## Data availability statement

The original contributions presented in the study are included in the article/**Supplementary Material**. Further inquiries can be directed to the corresponding author.

## Ethics statement

Written informed consent was obtained from the individual(s) for the publication of any potentially identifiable images or data included in this article.

## Author contributions

QZ extracted data, analyzed relevant information, and drafted the manuscript. YX, YF, and XC analyzed the data. LG and FL revised the manuscript. XZ designed the article. All authors contributed to the article and approved the submitted version.

## References

[1] Wei JN Sung FC Lin CC Lin RS Chiang CC Chuang LM. National surveillance for type 2 diabetes mellitus in Taiwanese children. JAMA (2003) 290(10):1345–50. doi: 10.1001/jama.290.10.1345 12966126

[2] Lone S Lone K Khan S Pampori RA. Assessment of metabolic syndrome in kashmiri population with type 2 diabetes employing the standard criteria's given by WHO, NCEPATP III and IDF. J Epidemiol Glob Health (2017) 7(4):235–9. doi: 10.1016/j.jegh.2017.07.004 PMC7384570 29110863

[3] Li F Du H Li S Liu. J. The association between metabolic syndrome and gastric cancer in Chinese. Front Oncol (2018) 8(326). doi: 10.3389/fonc.2018.00326 PMC6116659 30191141

[4] Alberti KG Eckel RH Grundy SM Zimmet PZ Cleeman JI Donato KA. Harmonizing the metabolic syndrome: a joint interim statement of the international diabetes federation task force on epidemiology and prevention; national heart, lung, and blood institute; American heart association; world heart federation; international atherosclerosis society; and international association for the study of obesity. Circulation (2009) 120(16):1640–5. doi: 10.1161/CIRCULATIONAHA.109.192644 19805654

[5] Lammert F Gurusamy K Ko CW Miquel JF Méndez-Sánchez N Portincasa P. Gallstones. Nat Rev Dis Primers (2016) 2:16024. doi: 10.1038/nrdp.2016.24 27121416

[6] Portincasa P Moschetta A Palasciano G. Cholesterol gallstone disease. Lancet (2006) 368(9531):230–9. doi: 10.1016/S0140-6736(06)69044-2 16844493

[7] Wirth J Joshi AD Song M Lee DH Tabung FK Fung TT. A healthy lifestyle pattern and the risk of symptomatic gallstone disease: results from 2 prospective cohort studies. Am J Clin Nutr (2020) 112(3):586–94. doi: 10.1093/ajcn/nqaa154 PMC7458768 32614416

[8] Attili. AF. Diabetes and gallstones. Dig Liver Dis (2011) 43(9):672–3. doi: 10.1016/j.dld.2011.06.018 21783441

[9] Bozkurt B Aguilar D Deswal A Dunbar SB Francis GS Horwich T. Contributory risk and management of comorbidities of hypertension, obesity, diabetes mellitus, hyperlipidemia, and metabolic syndrome in chronic heart failure: a scientific statement from the American heart association. Circulation (2016) 134(23):e535–78. doi: 10.1161/CIR.0000000000000450 27799274

[10] Zhu Q Sun X Ji X Zhu L Xu J Wang C. The association between gallstones and metabolic syndrome in urban han Chinese: a longitudinal cohort study. Sci Rep (2016) 6:29937. doi: 10.1038/srep29937 27443986 PMC4957232

[11] Nervi F Miquel JF Alvarez M Ferreccio C Garcia-Zattera MJ Gonzalez R. Gallbladder disease is associated with insulin resistance in a high risk Hispanic population. J Hepatol (2006) 45(2):299–305. doi: 10.1016/j.jhep.2006.01.026 16516330

[12] Mendez-Sanchez N Chavez-Tapia NC Motola-Kuba D Sanchez-Lara K Ponciano-Rodriguez G Baptista H. Metabolic syndrome as a risk factor for gallstone disease. World J Gastroenterol (2005) 11(11):1653–7. doi: 10.3748/wjg.v11.i11.1653 PMC4305948 15786544

[13] Sekula P Del Greco MF Pattaro C Kottgen A. Mendelian randomization as an approach to assess causality using observational data. J Am Soc Nephrol (2016) 27(11):3253–65. doi: 10.1681/ASN.2016010098 PMC5084898 27486138

[14] Smith GD Ebrahim S. Mendelian randomization: prospects, potentials, and limitations. Int J Epidemiol (2004) 33(1):30–42. doi: 10.1093/ije/dyh132 15075143

[15] Smith GD Ebrahim S. Mendelian randomization': can genetic epidemiology contribute to understanding environmental determinants of disease? Int J Epidemiol (2003) 32(1):1–22. doi: 10.1093/ije/dyg070 12689998

[16] Morrison J Knoblauch N Marcus JH Stephens M He X. Mendelian randomization accounting for correlated and uncorrelated pleiotropic effects using genome-wide summary statistics. Nat Genet (2020) 52(7):740–7. doi: 10.1038/s41588-020-0631-4 PMC7343608 32451458

[17] Birney E. Mendelian randomization. Cold Spring Harb Perspect Med (2022) 12(4):a041302. doi: 10.1101/cshperspect.a041302 34872952 PMC9121891

[18] Boef AG Dekkers OM le Cessie S. Mendelian randomization studies: a review of the approaches used and the quality of reporting. Int J Epidemiol (2015) 44(2):496–511. doi: 10.1093/ije/dyv071 25953784

[19] van Walree ES Jansen IE Bell NY Savage JE de Leeuw C Nieuwdorp M. Disentangling genetic risks for metabolic syndrome. Diabetes (2022) 71(11):2447–57. doi: 10.2337/db22-0478 35983957

[20] Pierce BL Ahsan H Vanderweele TJ. Power and instrument strength requirements for mendelian randomization studies using multiple genetic variants. Int J Epidemiol (2011) 40(3):740–52. doi: 10.1093/ije/dyq151 PMC3147064 20813862

[21] Manning AK Hivert MF Scott RA Grimsby JL Bouatia-Naji N Chen H. A genome-wide approach accounting for body mass index identifies genetic variants influencing fasting glycemic traits and insulin resistance. Nat Genet (2012) 44(6):659–69. doi: 10.1038/ng.2274 PMC3613127 22581228

[22] Willer CJ Schmidt EM Sengupta S Peloso GM Gustafsson S Kanoni S. Discovery and refinement of loci associated with lipid levels. Nat Genet (2013) 45(11):1274–83. doi: 10.1038/ng.2797 PMC3838666 24097068

[23] Hemani G Zheng J Elsworth B Wade KH Haberland V Baird D. The MR-base platform supports systematic causal inference across the human phenome. Elife (2018) 7:e34408. doi: 10.7554/eLife.34408 29846171 PMC5976434

[24] Bowden J Davey G Smith S. Burgess. mendelian randomization with invalid instruments: effect estimation and bias detection through egger regression. Int J Epidemiol (2015) 44(2):512–25. doi: 10.1093/ije/dyv080 PMC4469799 26050253

[25] Bowden J Davey Smith G Haycock PC Burgess S. Consistent estimation in mendelian randomization with some invalid instruments using a weighted median estimator. Genet Epidemiol (2016) 40(4):304–14. doi: 10.1002/gepi.21965 PMC4849733 27061298

[26] Ong JS MacGregor S. Implementing MR-PRESSO and GCTA-GSMR for pleiotropy assessment in mendelian randomization studies from a practitioner's perspective. Genet Epidemiol (2019) 43(6):609–16. doi: 10.1002/gepi.22207 PMC6767464 31045282

[27] Jiang L Du J Wang J Ding J. Sex-specific differences in the associations of metabolic syndrome or components with gallstone disease in Chinese euthyroid population. Sci Rep (2023) 13(1):1081. doi: 10.1038/s41598-023-28088-z 36658285 PMC9852245

[28] Kim Y Oh CM Ha E Park SK Jung JY Ryoo JH. Association between metabolic syndrome and incidence of cholelithiasis in the Korean population. J Gastroenterol Hepatol (2021) 36(12):3524–31. doi: 10.1111/jgh.15568 PMC9291184 34097775

[29] Despres JP Lemieux I Prud'homme D. Treatment of obesity: need to focus on high risk abdominally obese patients. BMJ (2001) 322(7288):716–20. doi: 10.1136/bmj.322.7288.716 PMC1119905 11264213

[30] Chen CH Huang MH Yang JC Nien CK Yang CC Yeh YH. Prevalence and risk factors of nonalcoholic fatty liver disease in an adult population of taiwan: metabolic significance of nonalcoholic fatty liver disease in nonobese adults. J Clin Gastroenterol (2006) 40(8):745–52. doi: 10.1097/00004836-200609000-00016 16940890

[31] Tung TH Ho HM Shih HC Chou P Liu JH Chen VT. A population-based follow-up study on gallstone disease among type 2 diabetics in kinmen, Taiwan. World J Gastroenterol (2006) 12(28):4536–40. doi: 10.3748/wjg.v12.i28.4536 PMC4125642 16874867

[32] Lim J Wirth J Wu K Giovannucci E Kraft P Turman C. Obesity, adiposity, and risk of symptomatic gallstone disease according to genetic susceptibility. Clin Gastroenterol Hepatol (2022) 20(5):e1083–120. doi: 10.1016/j.cgh.2021.06.044 PMC8720320 34217876

[33] Stender S Nordestgaard BG Tybjaerg-Hansen A. Elevated body mass index as a causal risk factor for symptomatic gallstone disease: a mendelian randomization study. Hepatology (2013) 58(6):2133–41. doi: 10.1002/hep.26563 23775818

[34] Zahor Z. Atherosclerosis in relation to cholelithiasis and cholesterolosis. Bull World Health Organ (1976) 53(5-6):531–7. PMC2366553 1087192

[35] Lin IC Yang YW Wu MF Yeh YH Liou JC Lin YL. The association of metabolic syndrome and its factors with gallstone disease. BMC Fam Pract (2014) 15:138. doi: 10.1186/1471-2296-15-138 25070766 PMC4118643

[36] Amigo L Zanlungo S Mendoza H Miquel JF Nervi F. Risk factors and pathogenesis of cholesterol gallstones: state of the art. Eur Rev Med Pharmacol Sci (1999) 3(6):241–6. 11261734

[37] Lioudaki E Ganotakis ES Mikhailidis DP. Lipid lowering drugs and gallstones: a therapeutic option? Curr Pharm Des (2011) 17(33):3622–31. doi: 10.2174/138161211798220909 22074432

[38] Nakeeb A Comuzzie AG Al-Azzawi H Sonnenberg GE Kissebah AH Pitt HA. Insulin resistance causes human gallbladder dysmotility. J Gastrointest Surg (2006) 10(7):940–8; discussion 948-9. doi: 10.1016/j.gassur.2006.04.005 16843864

