# Supplementary material for: Causal association between metabolic syndrome and cholelithiasis: a Mendelian randomization study
Source: Front Endocrinol (Lausanne). 2023 Jun 9;14:1180903. doi: 10.3389/fendo.2023.1180903 (PMC10288183; doi:10.3389/fendo.2023.1180903)
Supplement: Supplementary file 1 [file DataSheet_1.pdf]

Supplementary Figure S1: The Funnel plots for MetS and its components

Supplementary Figure S2: The Leave one out plots for MetS and its components

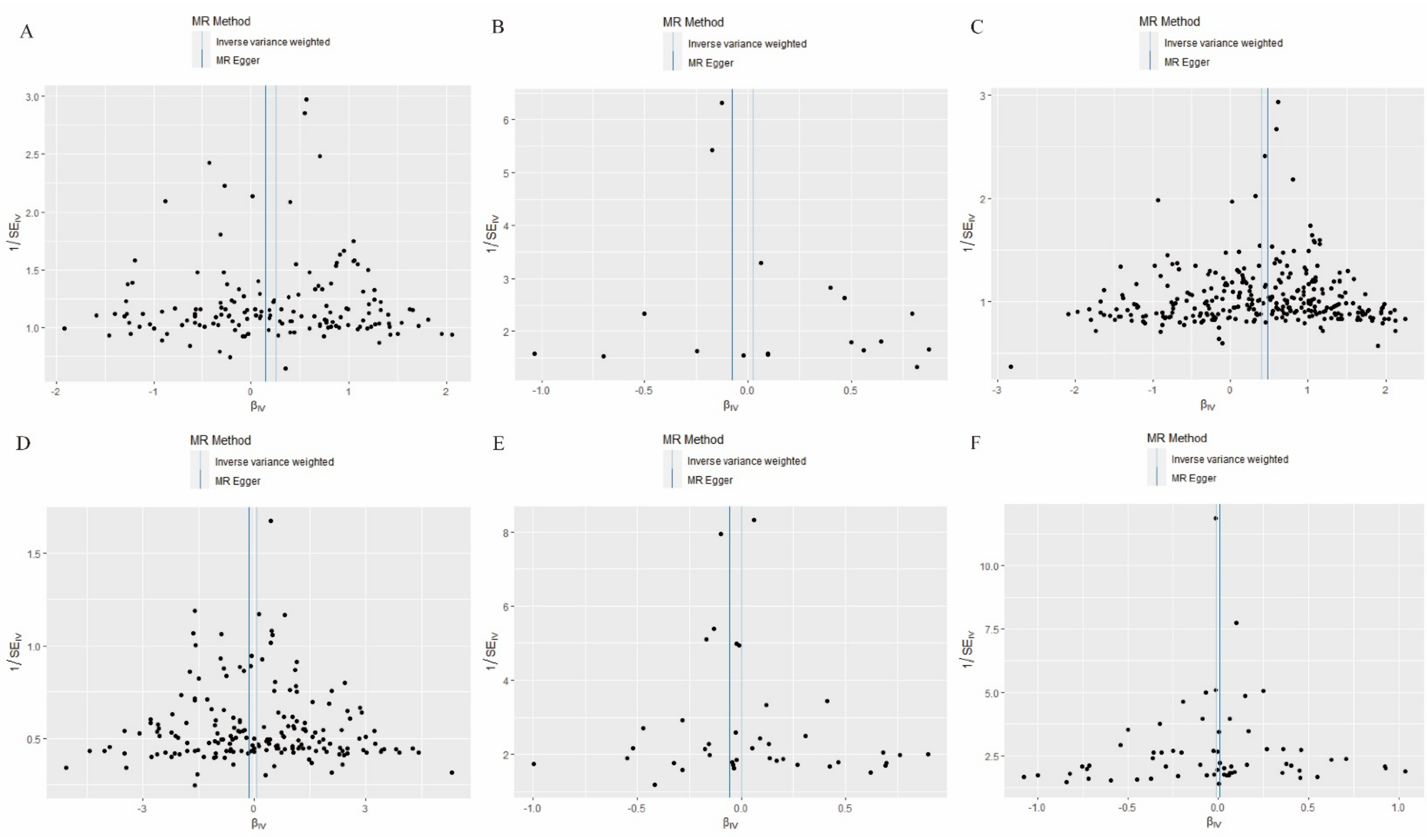

Supplementary Figure S1: The Funnel plots for MetS and its components. (A) Funnel plot of MetS and cholelithiasis; (B) Funnel plot of FBG and cholelithiasis; (C) Funnel plot of WC and cholelithiasis; (D) Funnel plot of hypertension and cholelithiasis; (E) Funnel plot of TG and cholelithiasis; (F) Funnel plot of HDL-C and cholelithiasis; MetS metabolic syndrome, FBG fasting blood glucose, WC waist circumference, TG triglycerides, HDL-C high-density lipoprotein cholesterol.

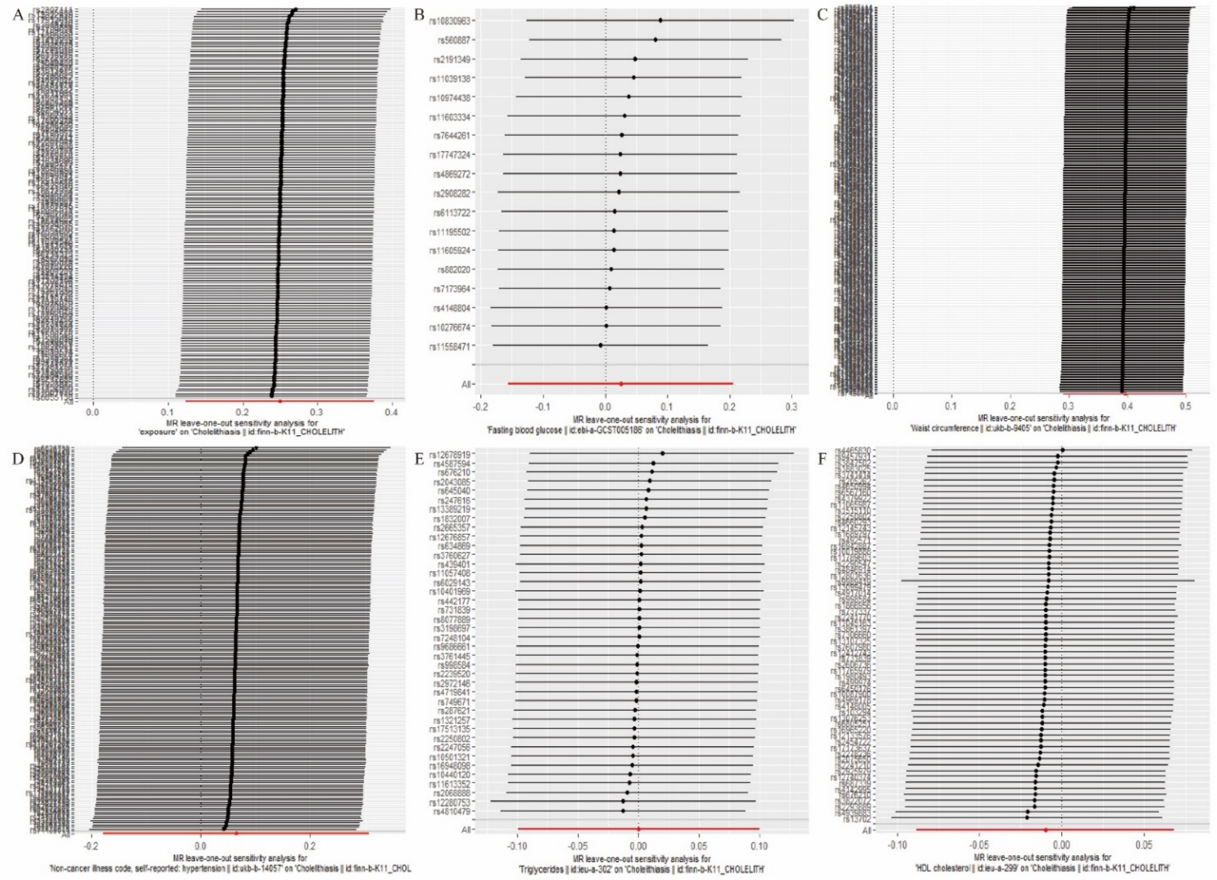

Supplementary Figure S2: The Leave one out plots for MetS and its components. (A) Leave one out plot of MetS and cholelithiasis; (B) Leave one out plot of FBG and cholelithiasis; (C) Leave one out plot of WC and cholelithiasis; (D) Leave one out plot of hypertension and cholelithiasis; (E) Leave one out plot of TG and cholelithiasis; (F) Leave one out plot of HDL-C and cholelithiasis; MetS metabolic syndrome, FBG fasting blood glucose, WC waist circumference, TG triglycerides, HDL-C high-density lipoprotein cholesterol.
